# Thanks to reviewers and editorial office

**DOI:** 10.1007/s40037-022-00737-5

**Published:** 2022-12-08

**Authors:** Lauren A. Maggio, Erik W. Driessen

**Affiliations:** 1 Department of Medicine, Uniformed Services University of the Health Sciences, Bethesda, MD USA; 2 School of Health Professions Education, Faculty of Health, Medicine and Life Science, Maastricht University, Maastricht, The Netherlands

It is an important tradition to thank our reviewers in our December issue. The reviewers’ critical and constructive feedback greatly contributes to ensuring the quality of Perspectives on Medical Education (PME). We recognize that health professions education is a multidisciplinary field and that its scholars are invited to review for many different journals likely multiple times per month. We also acknowledge that the reviewer pool is limited because we look for the right reviewer for a manuscript’s topic, method, and scientific tradition. Therefore, we’re incredibly grateful to all the reviewers for their precious time and valuable advice which helped us to assess and improve the more than 400 manuscripts submitted in 2022.

This year, we also want to thank PME’s editorial office. The people who work backstage to run the journal: Lieda Meester (our managing editor), Helen Dupuis (our language editor), Zephaniah Paulyn Reyes (our editorial office assistant) and Anja Heida (our publisher). A peer review journal is a logistically complex enterprise. Manuscripts move from authors to deputy editors, associate editors, reviewers, and the deputy editor-in-chief and editor-in-chief. The editorial office manages the exchange of the manuscripts between these individuals and tries to do so as quickly as possible. They are the people who send you the gentle reminders when you’re late with your review, revisions, or decision. The logistics is only one facet of the diversity of the editorial’s office work. Other facets are issue building; copy editing; answering questions from authors, reviewers, and editors; and advising editors on these topics. The language editor works with the editor and the authors to make the article’s language more clear, coherent, and accessible.

In 2023, PME will move to a new publisher: Ubiquity Press (https://pmejournal.org/). We look forward to this move, but it also means that we must take leave of our amazing editorial office team. We do this with a sad heart. We are deeply grateful for everything they have done for PME.

